# Identification of prognostic genes in the acute myeloid leukemia microenvironment

**DOI:** 10.18632/aging.102477

**Published:** 2019-11-18

**Authors:** Shaoxin Huang, Biyu Zhang, Wenyan Fan, Qihan Zhao, Lei Yang, Wang Xin, Denggang Fu

**Affiliations:** 1 School of Basic Medicine, Jiujiang University, Jiujiang, Jiangxi 332005, China; 2 School of Pharmacy and Life Science, Jiujiang University, Jiujiang, Jiangxi 332005, China; 3 Key Laboratory of System Bio-medicine of Jiangxi Province, Jiujiang University, Jiujiang, Jiangxi 332000, China; 4 Institute of Genomic and Personalized Medicine, School of Life Science and Technology, Huazhong University of Science and Technology, Wuhan, Hubei 430074, China

**Keywords:** acute myeloid leukemia, microenvironment, immune scores, stromal scores, overall survival

## Abstract

The tumor microenvironment (TME) has a strong influence on the progression, therapeutic response, and clinical outcome of acute myeloid leukemia (AML), one of the most common hematopoietic malignancies in adults. In this study, we identified TME-related genes associated with AML prognosis. Gene expression profiles from AML patients were downloaded from TCGA database, and immune and stromal scores were calculated using the ESTIMATE algorithm. Immune scores were correlated with clinical features such as FAB subtypes and patient’s age. After categorizing AML cases into high and low score groups, an association between several differentially expressed genes (DEGs) and overall survival was identified. Functional enrichment analysis of the DEGs showed that they were primarily enriched in the immune response, inflammatory response, and cytokine activity, and were involved in signaling processes related to hematopoietic cell lineage, B cell receptor, and chemokine pathways. Two significant modules, dominated respectively by CCR5 and ITGAM nodes, were identified from the PPI network, and 20 hub genes were extracted. A total of 112 DEGs correlated with poor overall survival of AML patients, and 11 of those genes were validated in a separate TARGET-AML cohort. By identifying TME-associated genes, our findings may lead to improved prognoses and therapies for AML.

## INTRODUCTION

Acute myeloid leukemia (AML) is one of the most prevalent and aggressive blood cancers in adults, accounting for about 1% of all cancers [1–3]. In the United States, an estimated 21,450 new cases and 10,920 deaths are projected to occur in 2019 [4]. AML is characterized by accumulation of immature myeloid hematopoietic cells, especially in the bone marrow. Peripheral blood involvement is also frequent, and may lead to malignant infiltration into the skin, lymph nodes, spleen, liver, and central nervous system [5]. The main therapeutic strategy for AML, i.e. intensive induction chemotherapy and postremission therapy, has remained basically unchanged for the last 30 years, without substantial improvement in patient survival [6, 7]. Although remarkable remissions can be initially attained through chemotherapy in most AML patients, complete disease elimination remains rare. Promising approaches have been proposed, such as chimeric antigen receptor (CAR) T-cell therapy targeting CD33 combined with allogeneic hematopoietic cell transplantation [8, 9]. However, 75% of patients are still at risk of disease relapse and succumb to the disease within 5 years from diagnosis [10].

AML prognosis is currently determined by increasing age, white blood cell counts at diagnosis, cytogenetic abnormalities, and AML-specific molecular genetic lesions [11, 12]. Although extensive research has helped to elucidate the genomic landscape of AML and to better understand its development, translation of this knowledge into improved therapies has just begun. Therefore, identification of potential biomarkers would aid in diagnosis, treatment, and prognosis of AML patients.

Much attention has been devoted in recent years to the role of the tumor microenvironment (TME) in cancer development [13]. Consequently, alterations in TME components have been defined in virtually all cancer types for each step of the multi-stage process of malignant progression, helping to understand cancer progression and to identify potential therapeutic targets [2]. For instance, diverse TME elements, including soluble factors, suppressive immune cells, and altered components of the extracellular matrix were shown to function together to restrain tumor immunotherapy, induce chemoresistance, and promote progression of breast cancer [14]. Likewise, breakthrough discoveries leading to current PD-1/PD-L1-targeted immunotherapies were the result of investigations assessing tumor-stromal interactions and specific alterations in the TME [15]. The tumor microenvironment has been revealed as a crucial determinant of the diagnosis and therapeutic response of cancer patients [2, 16–18]. The high complexity of the TME is reflected by multiple interactions between tumor, stromal, immune, and mesenchymal cells, through a number of soluble factors and alterations in extracellular matrix components [19]. As the two major non-tumor cell populations in the TME, stromal cells and infiltrating immune cells have been associated with tumor diagnosis and prognosis. For instance, analysis of RNA-seq gene expression data showed that immune infiltration by T and B cells, including increased abundance of CD8+ T cells and B-cell receptor diversity, is associated with improved overall survival in Merkel cell carcinoma [20]. Indeed, the TME is considered a consensus field for identifying novel tumor biomarkers [21, 22]. Since the interplay between leukemic blasts and the bone marrow microenvironment has shown to affect chemotherapy resistance in AML, targeting the TME interactions in AML has been the focus of several preclinical studies and early phase clinical trials [23, 24]. Examples include inhibitors of CXCR4 [25, 26], VLA-4 [27, 28] and E-selectin [29], which are being evaluated in clinical trials.

Algorithms that evaluate and rank immune and stromal populations within the TME, such as the Estimate of STromal and Immune cells in MAlignant Tumor tissues using Expression data (ESTIMATE) [30], have been developed to assess the infiltration of non-tumor cells by analyzing specific gene expression signatures [31]. Although this algorithm has been successfully applied to characterize several solid tumors, such as breast cancer [32], clear cell renal cell carcinoma [33], and colon cancer [34], it has not been used to define immune and stromal scores in AML samples.

In the present study, gene expression profiles and clinical information of AML cohorts were downloaded from TCGA, and the ESTIMATE algorithm was then used to calculate immune and stromal scores for these AML cases. Following classification into high- and low-score groups, we identified a subset of TME-associated genes that predict outcome in patients with AML. The correlation between the expression of these genes and AML prognosis was independently validated in a TARGET AML cohort from UCSC Xena database. These findings may contribute to better understand the role of the TME in AML and might lead to improved prognosis and therapies for this disease.

## RESULTS

### Correlation between immune and stromal scores and AML clinical parameters

**Figure 1:**
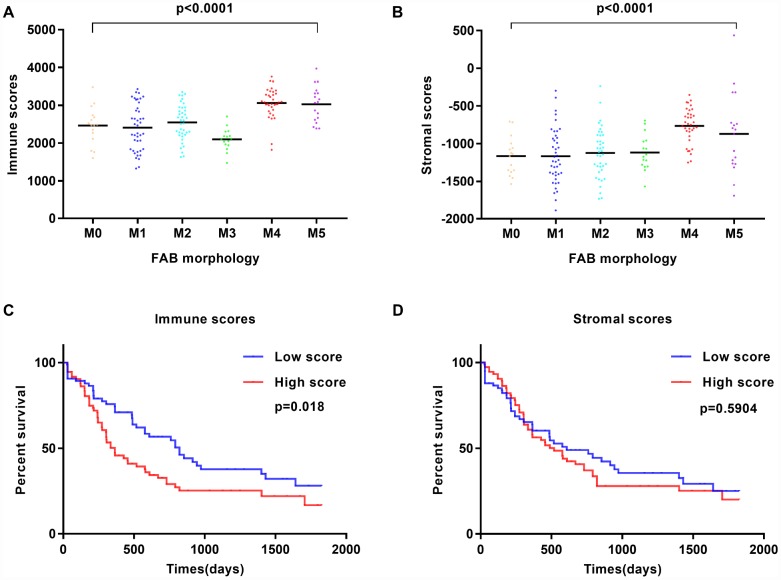
**Immune scores and stromal scores are associated with AML FAB subtypes and overall survival.** (**A**) Distribution of immune scores within AML FAB subtypes. Significant associations were detected between AML FAB subtypes and immune scores (n = 173, p < 0.0001). (**B**) Distribution of stromal scores within AML FAB subtypes. Significant associations were found between AML FAB subtypes and stromal scores (n = 173, p < 0.0001). (**C**) Kaplan-Meier survival analysis of high vs. low immune score groups (log-rank test, p = 0.018). (**D**) Kaplan-Meier survival analysis of high vs. low stromal score groups (log-rank test, p = 0.5904).

Gene expression profiles and associated clinical data of 173 AML patients were retrieved from TCGA database. Among patients, 80 (46.2%) were female and 93 (53.8%) were male. Pathological diagnosis identified 16 cases of FAB M0 (undifferentiated subtype), 42 FAB M1 cases, 39 FAB M2 cases, 16 FAB M3 cases, 35 FAB M4 cases, and 19 cases of FAB M5 (Table 1). Immune scores and stromal scores for these patients were calculated using the ESTIMATE algorithm. Immune scores ranged from 1329.53 to 3971.97, whereas stromal scores varied from -1888.81 to 435.75. The relationship between immune and stromal scores and clinical parameters was analyzed. On average, immune scores of FAB M4 cases ranked the highest among all 6 FAB morphological subtypes, while immune scores from FAB M3 patients ranked the lowest (p < 0.001; Figure 1A). Similarly, FAB M4 cases had the highest stromal scores, whereas FAB M0 and M1 subtypes had the lowest (p < 0.0001; Figure 1B). No significant correlations between immune or stromal scores and patients’ gender or history of neoadjuvant treatment were observed using two-tailed Student’s t-tests, while immune scores showed a positive association with both cytogenetic risk category and age (Supplementary Figure 1). These findings suggest that the analysis of immune and stromal scores may aid in the diagnosis and characterization of specific AML subtypes.

**Table 1:** The clinical data of patients with AML based on the immune scores and stromal scores.

**Characteristic**	**Immune score(range)**	**Stromal score(range)**	**Cases**
**Age**			
<60	(1368.53-3758.87)	(-1888.81--202.78)	90
≥60	(1329.53-3971.97)	(-1753.86-435.75)	83
**Gender**			
Female	(1329.53-3971.97)	(-1888.81-435.75)	80
Male	(1475.85-3758.87)	(-1660.43- -207.13)	93
**Neoadjuvant treatment**			
Yes	(1329.53-3971.97)	(-1888.81-435.75)	45
No	(1475.85-3758.87)	(-1735.4- -207.13)	128
**FAB subtype**			
M0	(1606.38-3481.5)	(-1534.51- -705.43)	16
M1	(1329.53-3432.53)	(-1888.81- -297.73)	42
M2	(1637.44- 3352.15)	(-1735.4- -235.75)	39
M3	(1475.85-2707.91)	(-1571.73-1734.11)	16
M4	(1823.93-3758.87)	(-1249.43- -352.55)	35
M5	(2388.6-3971.97)	(-1693.75-435.75)	18
M6	(2698.17-3116.63)	(-754.82- -494.19)	2
M7	(1970.08-2489.15)	(-1094.44- -207.18)	3
Unkown	(1625.8-2150.49)	(-1462.53- -1398.47)	2
**Cytogenetics_risk_category**			
Favorable	(1475.85-3758.87)	(-1571.73- -425.53)	32
Normal	(1329.53-3971.97)	(-1753.86-435.75)	103
Poor	(1625.8-3481.5)	(-1888.81- -235.75)	36
NA	(1839.34-2510.82)	(-1735.4- -1470.38)	2
**Survival status**			
Alive	(1329.53-3758.87)	(-1888.81- -202.78)	70
Dead	(1579.75-3971.97)	(-1735.4- 435.75)	103

*AML: Acute Myeloid Leukaemia; FAB: French-American-British.

To assess the correlation between immune and stromal scores and overall survival, AML patients were divided into corresponding high- and low-score groups. We found that cases with a low immune score had significantly longer overall survival than those with a high immune score (p = 0.018; Figure 1C). Meanwhile, patients with low stromal scores showed longer overall survival than patients with high stromal scores, but this difference was not significant (p = 0.5904; Figure 1D). In addition, patients without history of chemotherapy had a better outcome than those treated with chemotherapy (Supplementary Figure 2B), while a favorable cytogenetic risk index predicted significantly improved prognosis (Supplementary Figure 2D). These observations suggest that patients with low immune and stromal scores have a more favorable outcome.

### Identification of differentially expressed genes

**Figure 2:**
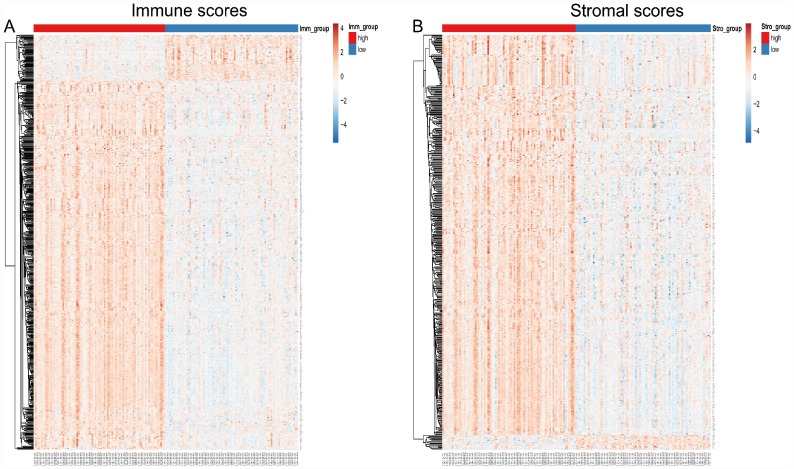
**Heatmap of differentially expressed genes in the high and low immune/stromal score groups.** (**A**) Immune scores (high score, left; low score, right. |log FC| > 1.5, q-value < 0.05). (**B**) Stromal scores (high score, left; low score, right. |log FC| > 1.5, q-value < 0.05).

**Figure 3:**
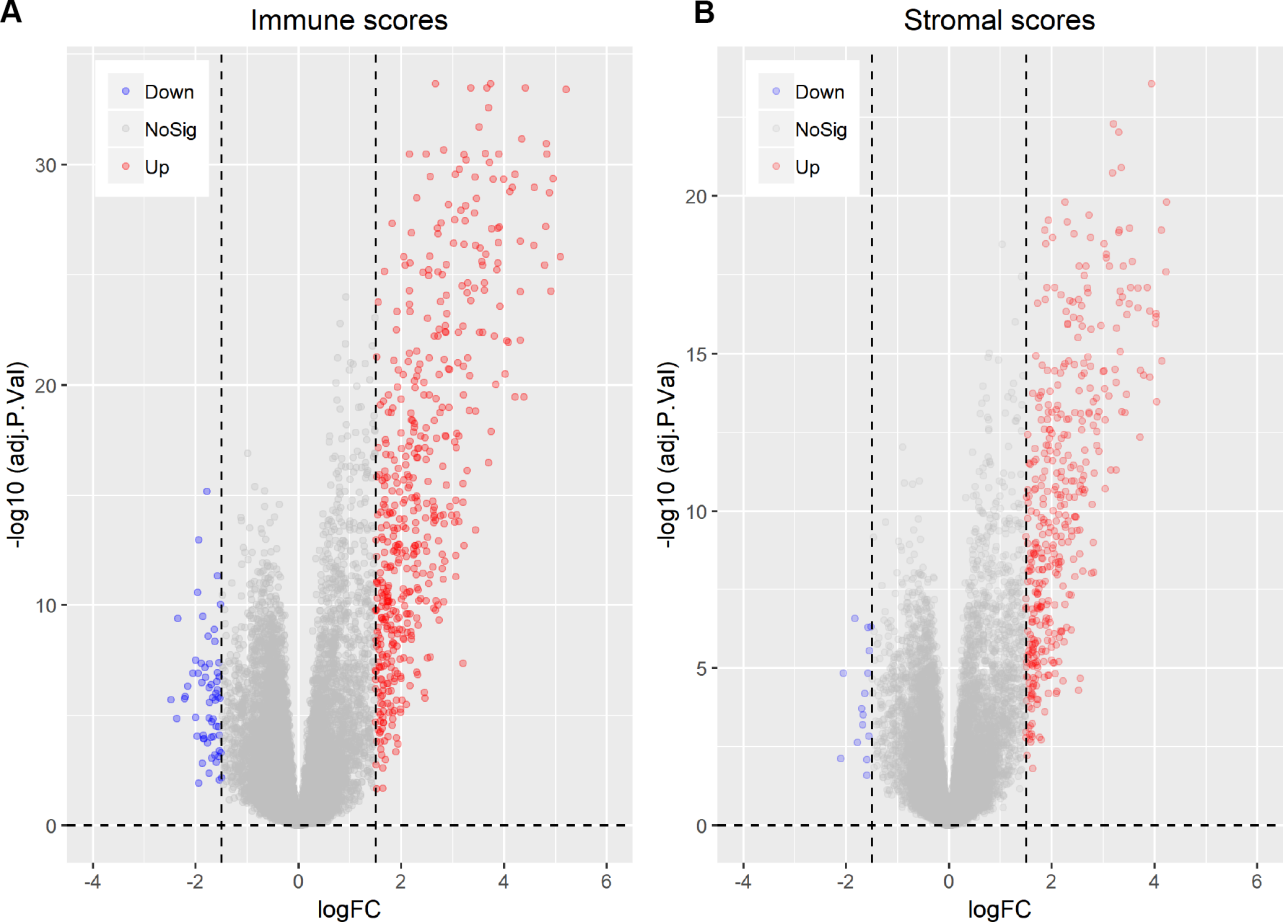
**Differentially expressed genes between high vs. low immune and stromal AML scores.** (**A**) Immune scores (|log FC| > 1.5, q-value < 0.05). (**B**) Stromal scores (|log FC| > 1.5, q-value < 0.05).

**Figure 4:**
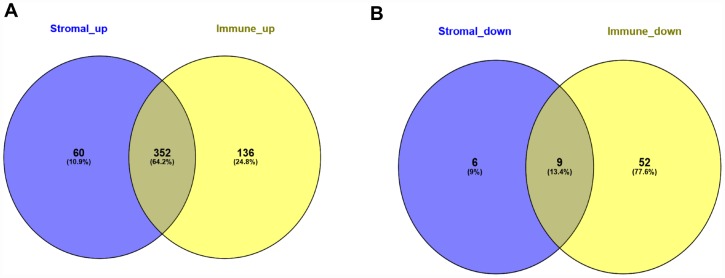
**Common differentially expressed genes detected for immune and stromal scores.** (**A**) Commonly upregulated DEGs. (**B**) Commonly downregulated DEGs.

To correlate gene expression profiles with immune and stromal scores, gene expression data from the 173 AML patients was analyzed after separation into high- and low-score groups based on median scores. A distinct gene expression pattern was revealed between the respective high- and low-score groups for both immune and stromal scores (Figure 2), indicating that gene expression profiles might be used to delineate group differences. On comparison based on immune scores, 488 genes were upregulated while 61 genes were downregulated (|log FC| > 1.5, q-value < 0.05) (Figure 3A). In turn, 412 genes were upregulated and 15 genes were downregulated upon comparison between the high and low stromal score groups (|log FC| > 1.5, q-value < 0.05) (Figure 3B). In addition, commonly shared DEGs were analyzed in the high and low groups based on immune and stromal scores. A total of 352 genes were upregulated (Figure 4A) while 9 genes were downregulated (Figure 4B). The proportion of commonly upregulated and downregulated genes found upon comparison of high vs. low stromal score groups was similar. These 361 genes were selected as DEGs for subsequent analysis to explore their relevance in association with the AML microenvironment.

### Functional enrichment analysis of DEGs

**Figure 5:**
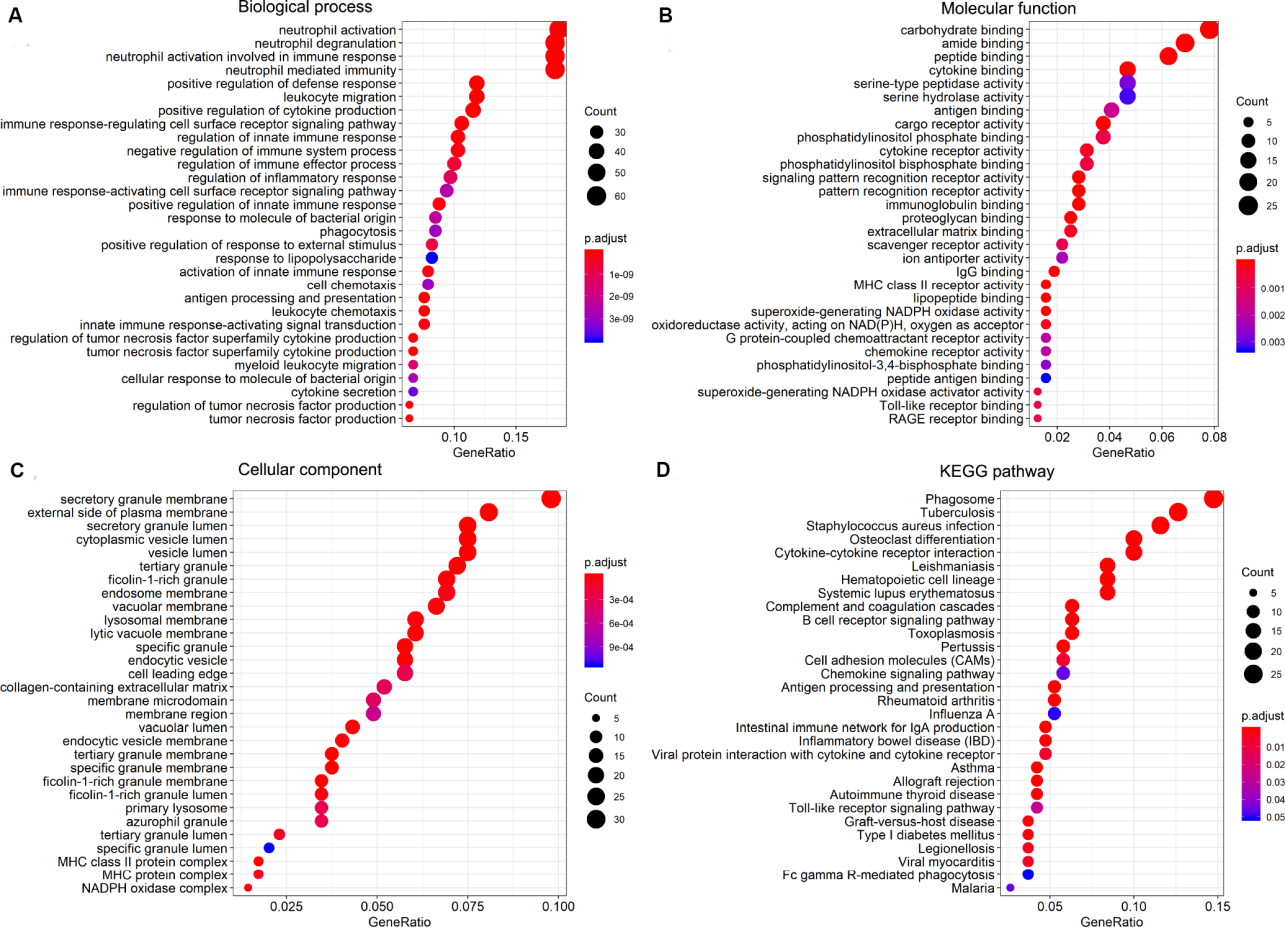
**GO term and KEGG pathway enrichment analysis of the top 30 DEGs.** (**A**) Biological process (BP). (**B**) Molecular function (MF). (**C**) Cellular component (CC). (**D**) KEGG pathway analysis.

To investigate the potential function of the DEGs identified above, GO term and KEGG pathway enrichment analyses were performed using the clusterProfiler package. Significance (false discovery rate < 0.05) was achieved for a total of 531 GO terms of biological process, 64 GO terms of molecular function, and 61 GO terms of cellular component. The top 30 GO biological process terms indicated that the DEGs were primarily enriched in ‘regulation of immune response process’, ‘activity of neutrophils and leukocytes’, ‘cytokine secretion’, ‘inflammatory response’, and ‘regulation of tumor necrosis’ (Figure 5A). Molecular functions ascribed to these DEGs included mainly ‘peptide binding’, ‘cytokine binding’, ‘immunoglobulin binding’, ‘lipopeptide binding’, and several sub-terms of ‘cargo receptor activity’ (Figure 5B). Primary terms within cellular component included ‘secretory granule membrane’ and ‘secretory granule lumen’, and ‘vesicle lumen’ (Figure 5C). Additionally, on KEGG analysis the DEGs were mainly enriched in ‘infection’, ‘hematopoietic cell lineage’, ‘B cell receptor signaling’ and ‘chemokine signaling’ pathways (Figure 5D). These analyses suggest a vital role for these DEGs in AML development, and merit further investigation to define their true biological contribution.

### Protein-protein interaction network

**Figure 6:**
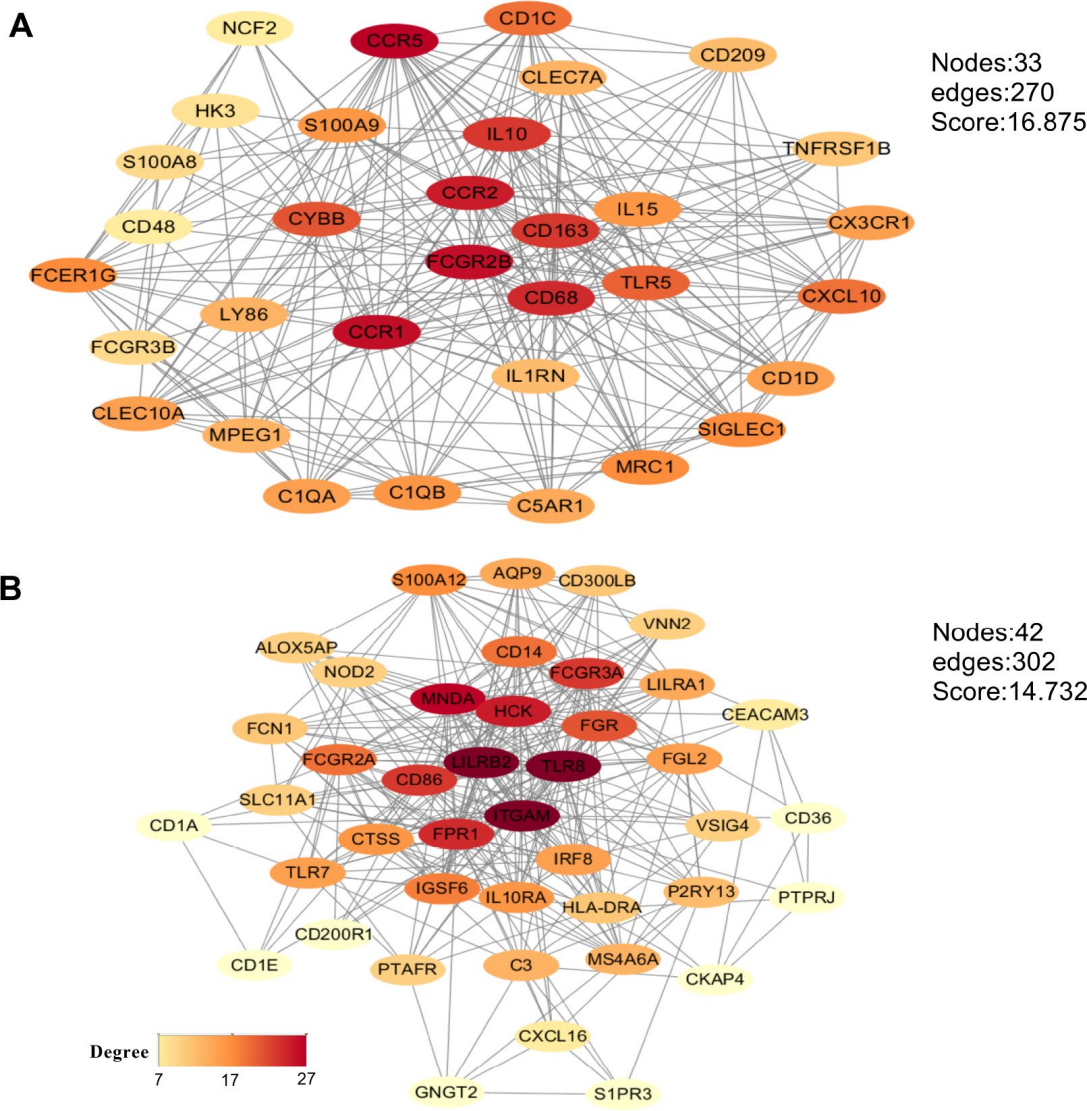
**Main modules identified through protein-protein interaction network analysis.** (**A**) CCR5 module. (**B**) ITGAM module. The color of a node in each module reflects its connectivity degree score.

To analyze potential connectivity patterns between the transcripts of our DEG set, a protein-protein interaction (PPI) network was constructed using the STRING database. The network revealed two significant modules (Figure 6). We called these modules CCR5 (chemokine receptor 5) and ITGAM (integrin alpha M), in reference to the highest-degree genes within each module. The CCR5 module (Figure 6A) was defined by 270 edges involving 33 nodes. CCR5, CCR1, FCGR2B, CCR2, CD68, CD163, and IL10 were the top 20% degree ranked nodes. Meanwhile, in the ITGAM module (Figure 6B), ITGAM, TLR8, LILRB2, MNDA, HCK, FPR1, CD86, and FCGR3A were the nodes with highest connectivity. After loading the entire PPI network on Cytoscape, the top 20 high-degree hub genes were identified using the cytoHubba plugin (Supplementary Table 1). These included all the top proteins identified in the CCR5 and ITGAM modules. Of note, most of these key nodes consisted of proteins/genes involved in immune regulation.

### Association between individual DEGs and overall AML survival

**Figure 7:**
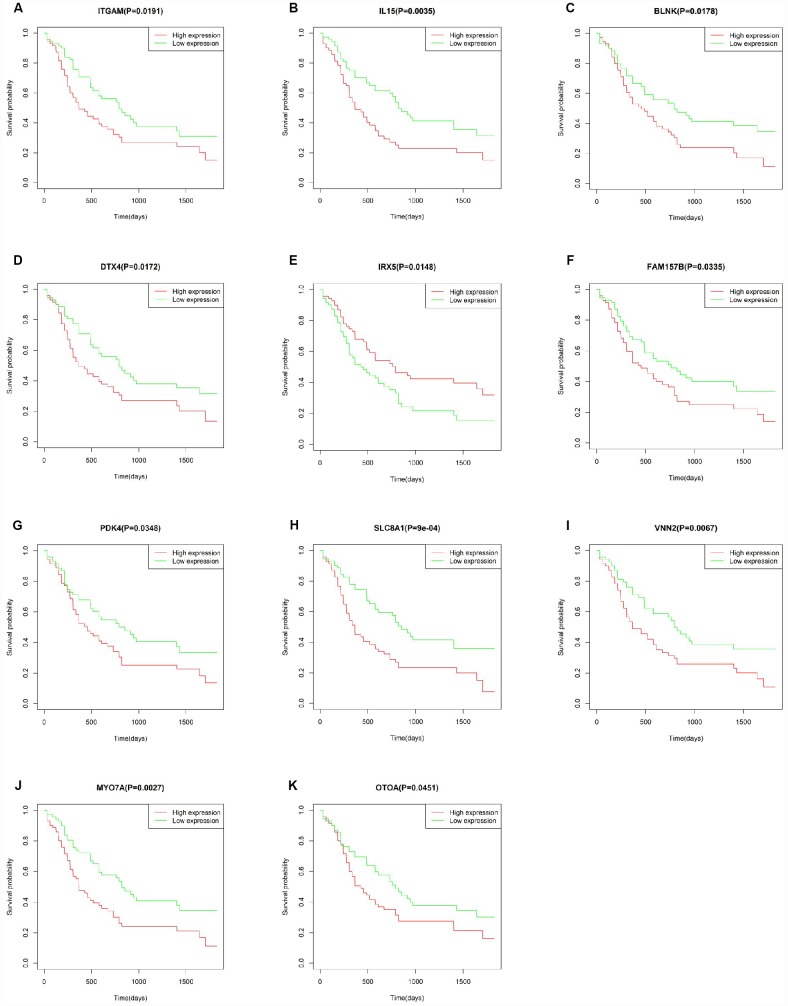
**Correlation of the expression of individual DEGs with overall survival of AML patients from TCGA database.** (**A**–**K**) Kaplan-Meier survival curves for selected DEGs following comparison of high vs. low gene expression groups according to the median value of each gene (log-rank test, p < 0.05).

**Figure 8:**
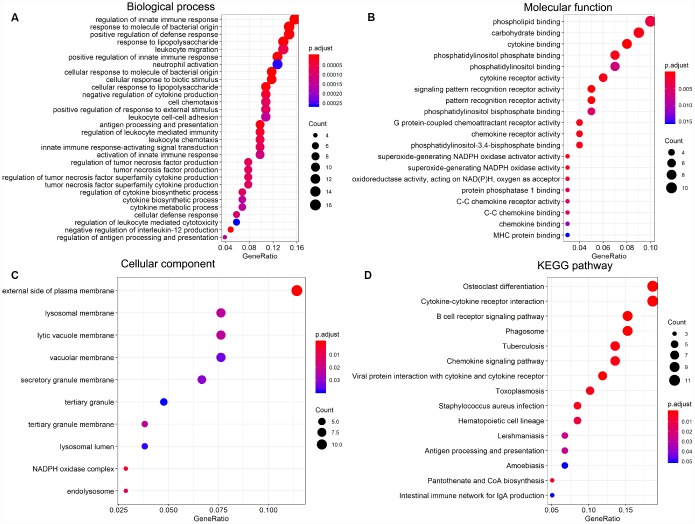
**Significantly enriched GO terms and KEGG pathways of prognosis-predictive DEGs from AML samples.** (**A**) Biological process (BP). (**B**) Molecular function (MF). (**C**) Cellular component (CC). (**D**) KEGG pathway analysis.

To explore the prognostic utility of individual DEGs on overall AML survival, Kaplan-Meier (K-M) survival curves were generated by the survival package in R. In total, 112 genes (a full list is shown in Supplementary Table 2), including 9 hub genes, were significantly correlated with poor overall survival using log-rank test (p < 0.05). K-M curves were plotted for several selected genes (Figure 7). GO analysis of these potential prognostic genes also showed strong association with the immune response, cytokine activity, chemotaxis, and leukocyte activation (Figure 8A–8C). Pathway analysis indicated that these genes were mainly involved in ‘cytokine-cytokine receptor interaction’, ‘B cell receptor signaling’, ‘chemokine signaling’, ‘hematopoietic cell lineage’, and ‘antigen processing and presentation’ (Figure 8D).

### Validation in the TARGET-AML cohort

**Figure 9:**
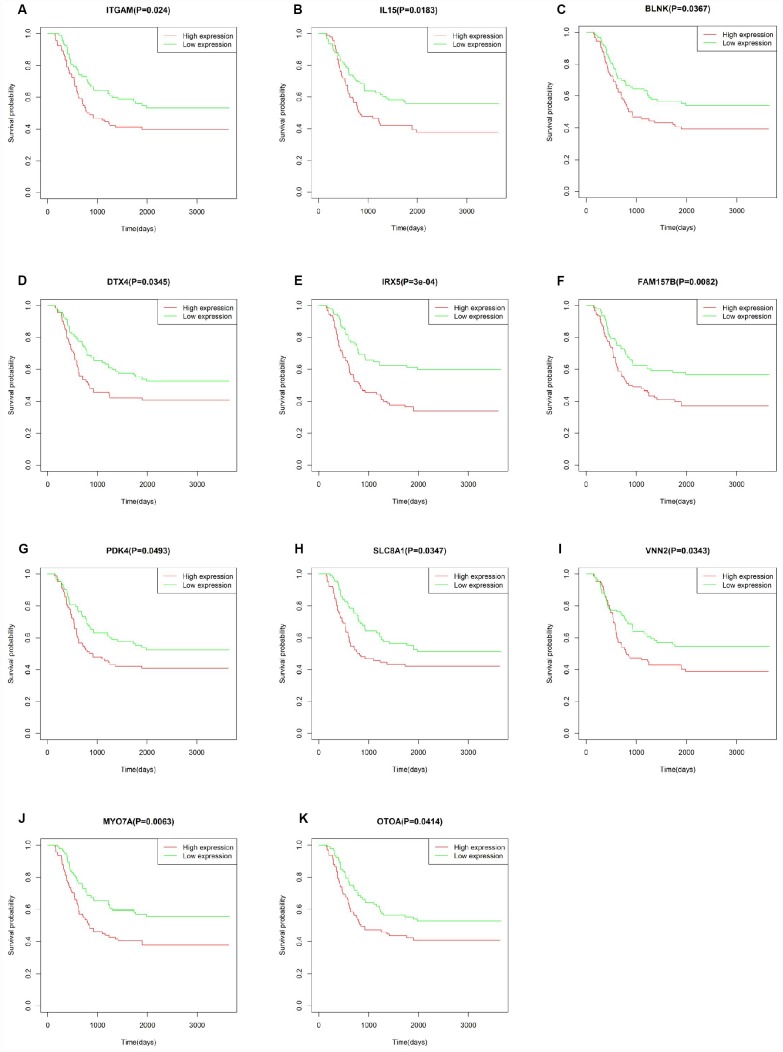
**Correlation of DEG expression with overall survival in the TARGET-AML dataset.** (**A**–**K**) Kaplan-Meier survival curves validating the correlation between 11 DEGs and overall survival in the TARGET-AML dataset (log-rank test p < 0.05; n = 187).

To verify whether the genes identified from TCGA AML patients are also of prognostic significance in an independent AML cohort, we downloaded and analyzed gene expression data of 187 TARGET AML patients from UCSC Xena database. Among 112 prognostic genes, a total of 11 genes were validated based on significant association with poor overall survival (Figure 9). Except for IRX5, the expression of all these validated genes showed a positive correlation with CXCR4 expression (Supplementary Figure 3), while ITGAM and SLC8A1 were negatively correlated with E-selectin and VLA-4 (Supplementary Figure 4). Similarly, a negative correlation between MYOTA and OTOA expression and E-selectin and VLA-4 expression was observed.

## DISCUSSION

**Figure 10:**
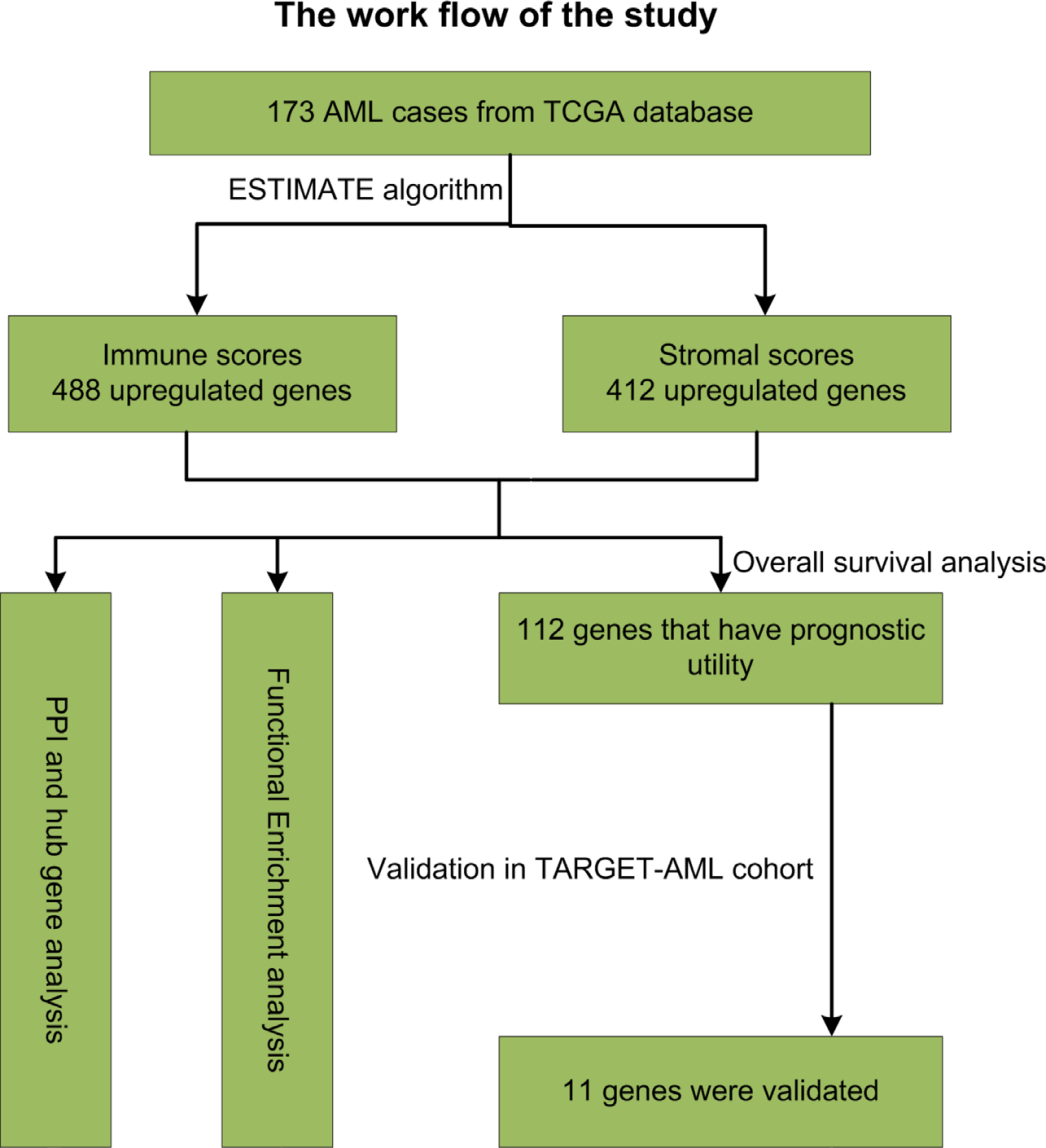
**Study workflow.**

The TME plays an essential role in the development, progression, and relapse of AML. Therefore, targeting the TME has become an effective tool to improve patient outcomes [2]. The main purpose of this study was to identify on the TCGA database TME genes that contribute to overall survival in AML patients. Following the general analysis workflow diagrammed in Figure 10, analysis of 173 AML cases identified 549 DEGs between high and low immune score patients, while 427 DEGs distinguished cases with high vs low AML stromal scores. We also detected an association between immune scores and diverse AML clinical parameters, including cytogenetic risk categories, older age, morphological FAB subtypes, and patient outcomes. Thus, we show that prognosis is worse for patients with a high immune score, while longer overall survival is predicted for cases with lower cytogenetic risk, no history of chemotherapy, FAB M3 subtype, or patients with age greater older than 60 years. These results were consistent with previous studies [34]. Further classification of cases revealed 361 common DEGs between high vs low immune/stromal score patients. GO analysis of these DEGs revealed significant enrichment in immune-related processes known to contribute to disease progression and drug resistance in several tumors [34, 35], including lung cancer [36], breast cancer [37] and bladder cancer [38]. Accordingly, KEGG analysis suggested the involvement of DEGs in several signaling pathways including ‘chemokine signaling pathway’ [39] and ‘intestinal immune network for IgA production’ [40], which may influence TME dynamics and development of AML. On the other hand, signal transduction pathways involving ‘cytokine-cytokine receptor interaction’ contribute to the progression of glioblastoma [41] and osteosarcoma [42], and modulate the microenvironment of hematopoietic tumors.

Hematopoietic stem cells on the endosteal surface of the bone marrow interact with a variety of cellular and extracellular components, such as osteoblasts, macrophages, and collagen and laminin fibers, and may act as progenitors for cancer-associated adipocytes and fibroblasts [43]. Importantly, these cells can also condition and reshape the TME, facilitating cancer cell proliferation, survival, chemotherapy resistance, and metastasis [43]. The interaction between hematopoietic cells and niche components plays a critical role in AML’s development, progression, survival, response to treatment, and relapse [23, 24, 44]. Previous studies showed that co-culture of AML blasts with bone marrow stromal cells stimulated blasts’ survival and inhibited chemotherapy-induced apoptosis, highlighting the critical role of the microenvironment with implications for chemotherapy and other treatment strategies for AML patients [45, 46].

We constructed a PPI network based on the 361 DEGs commonly shared between high vs low immune/stromal score patients, and identified two significant modules with genes primarily enriched in ‘immune/inflammatory response’, ‘chemokine binding’, and ‘myeloid leukocyte activation’. CCR5 and ITGAM were the top interrelated nodes in these two modules, and their expression predicted poor prognosis in our study. ITGAM (also known as CD11b) is a differentiation marker for cells of the myeloid-monocytic lineage [47]. Upregulation of ITGAM and CD86 following LSD1 inhibition was correlated with myeloid differentiation, inhibition of human monocytic leukemia cell proliferation [48], and sensitization of AML cells to all-trans-retinoic acid [49]. Meanwhile, CCR5 regulates proliferation and plays a key role in the extramedullary homing of infiltrating leukemia cells [3].

Correlation analysis of individual DEGs identified 112 genes, including 9/20 top hub genes in the PPI network, in association with poor overall AML survival. Functional enrichment analysis confirmed the involvement of these hub genes in immune-related processes. A separate cohort (TARGET-AML) of 187 AML cases from UCSC Xena database was used to validate the survival analysis. Results showed that 11 DEGs from our TCGA AML cohort were also significantly linked to poor overall survival in the TARGET-AML cohort. Most of the validated genes, such as IL15 [50], ITGAM [51], PDK4 [52, 53], and IRX5 [54], have been implicated in AML progression and/or as survival predictors in several types of cancers. The remaining genes, i.e. BLNK [55], MYO7A [56], VNN2 [57], OTOA [58], SLC8A1 [59] and DTX4 [60], might also serve as potential biomarkers for AML. CXCR4 and adhesion molecules such as E-selectin and VLA-4 have been targeted to develop clinical therapies. We found that all the validated genes, except for IRX5, are positively correlated with CXCR4 expression, whereas SLC8A1 and ITGAM showed a negative correlation with E-selectin and VLA4 expression. CXCR4 is expressed on the surface of AML blasts, and increased expression predicts poor survival and high relapse rate [61]. Accordingly, AML progression can be facilitated by interaction of CXCL12 and CXCR4 to activate the MEK/ERK and PI3K/AKT pathways [25]. The VCAM-1/VLA-4 pathway modulates interactions between hematopoietic stem cells within the bone marrow and with fibronectin or stromal cells to activate the PI3K/AKT/Bcl-2 signaling pathway, inducing resistance to chemotherapy [62]. Although experimental verification is warranted, the association of these genes suggested that they may promote the development of AML in a specific manner.

IL15 and ITGAM, two hub genes from the main PPI modules, are of particular interest. As a proinflammatory cytokine, IL15 has a variety of functions in the immune system and in the generation of multiple lymphocyte subsets [63], affecting the proliferation and differentiation of natural killer, T, and B cells [64], and CD8+ T memory cells [65]. In patients with colorectal carcinoma, absence of IL-15 expression correlated with decreased immune activation assessed by T and B cell abundance, and predicted worse prognosis [66]. There is evidence that IL15 can promote the pathogenesis of leukemia [67] and control the proliferation and survival of leukemic progenitors [68]. IL15 can induce survival and proliferation of growth factor-dependent AML cells through interaction with IL2 receptor beta/gamma [69], and its upregulation may be a predictor of disease relapse in pediatric AML patients [70]. Moreover, single nucleotide polymorphisms in the IL15 gene have been associated with risk of developing adult acute lymphoblastic leukemia [71]. These studies highlight the potential relevance of IL15 targeting in therapies for hematopoietic cancers.

ITGAM [72] was the highest interconnected node from the MCODE modules. ITGAM is a cell surface receptor selectively expressed on leukocytes with multifaceted functions in the activation, chemotaxis, cytotoxicity, phagocytosis, and interaction of leukemic cells with the TME [73, 74]. ITGAM is considered a marker for myeloid-derived suppressor cells responsible for tumor escape from host immunity and treatment refractoriness [75, 76]. Resistance to chemotherapy is a major obstacle in AML therapy. High ITGAM/CD56 co-expression combined with low Smac/DIABLO expression were proposed to be an important predictor of chemoresistance in AML patients [77]. The prognostic value of ITGAM in AML patients has been extensively assessed, and a correlation between high ITGAM expression and poor prognosis in AML has been established [51, 78]. Accordingly, ITGAM expression predicted worse overall survival in the present study.

## CONCLUSIONS

In summary, our study used the ESTIMATE algorithm to define a set of TME-related DEGs based on immune and stromal scores from TCGA AML data. Correlation analysis of the expression of these genes with patients’ overall survival was performed and results independently validated in the TARGET-AML cohort. Further studies on the DEGs identified here should help clarify the mechanisms by which gene expression within the TME influences the prognosis and progression of AML, and guide the development of more effective therapies.

## MATERIALS AND METHODS

### Gene expression datasets

Gene expression profiles of 173 AML patients were downloaded from TCGA database (https://portal.gdc.cancer.gov/). Clinical data, including gender, age, French-American-British (FAB) classification [79], history of neoadjuvant treatment, survival, and outcome, were also downloaded. Immune scores and stromal scores were calculated by the ESTIMATE algorithm. For validation of TCGA data, gene expression profiles of 187 TARGET-AML patients were obtained from the UCSC Xena database (https://xenabrowser.net/datapages/). Clinical follow-up information was also downloaded.

### Identification of differentially expressed genes

Differentially expressed genes (DEGs) were identified between high and low score groups stratified by the median value of immune scores and stromal scores using limma package [80]. Genes with |log FC |> 1.5 and adjusted p-value (q value) < 0.05 were selected as DEGs.

### Heatmap and clustering analysis

Heatmap and clustering were performed using the online tool ClustVis (https://biit.cs.ut.ee/clustvis/) [81].

### Enrichment analysis of DEGs

Functional enrichment analysis of DEGs was conducted by clusterProfiler [81] R package to identify GO categories, including biological processes (BP), molecular functions (MF), and cellular components (CC). Pathway enrichment analysis based on the Kyoto Encyclopedia of Genes and Genomes (KEGG) database was also performed using this package. P < 0.05 was considered statistically significant.

### Protein network construction and identification and analysis of hub genes

To explore potential relationships among DEGs, a protein-protein interaction (PPI) network was retrieved from the STRING database [82] and reconstructed using Cytoscape software [83]. The Molecular Complex Detection (MCODE) [84] plugin in Cytoscape was used to identify densely connected modules in the PPI network with the default parameters “Degree Cutoff = 2”, “Node Score Cutoff = 0.2”, “K-Core = 2” and “Max.Depth = 100”. Individual networks with 15 or more nodes were considered as significant modules. The top 20 hub genes were identified based on degree ranking using the cytoHubba plugin [85] in Cytoscape software.

### Overall survival analysis

The survival R package was used to analyze the relationship between the expression of DEGs (including hub genes) and patients’ overall survival using the log-rank test. In addition, Pearson’s correlations between expression data of the validated genes and CXCR4, VLA-4, and E-selectin expression were also obtained. P < 0.05 was considered statistically significant.

### Ethical approval

This article does not contain either human nor animal experiments.

## Supplementary Material

Supplementary Figures

Supplementary Tables

Supplementary Table 2
