# Supplementary material for: Identification of prognostic genes in the acute myeloid leukemia microenvironment
Source: Aging (Albany NY). 2019 Nov 18;11(22):10557–80. doi: 10.18632/aging.102477 (PMC6914404; doi:10.18632/aging.102477)
Supplement: Supplementary Figures [file aging-11-102477-s003..pdf]

SUPPLEMENTARY FIGURES

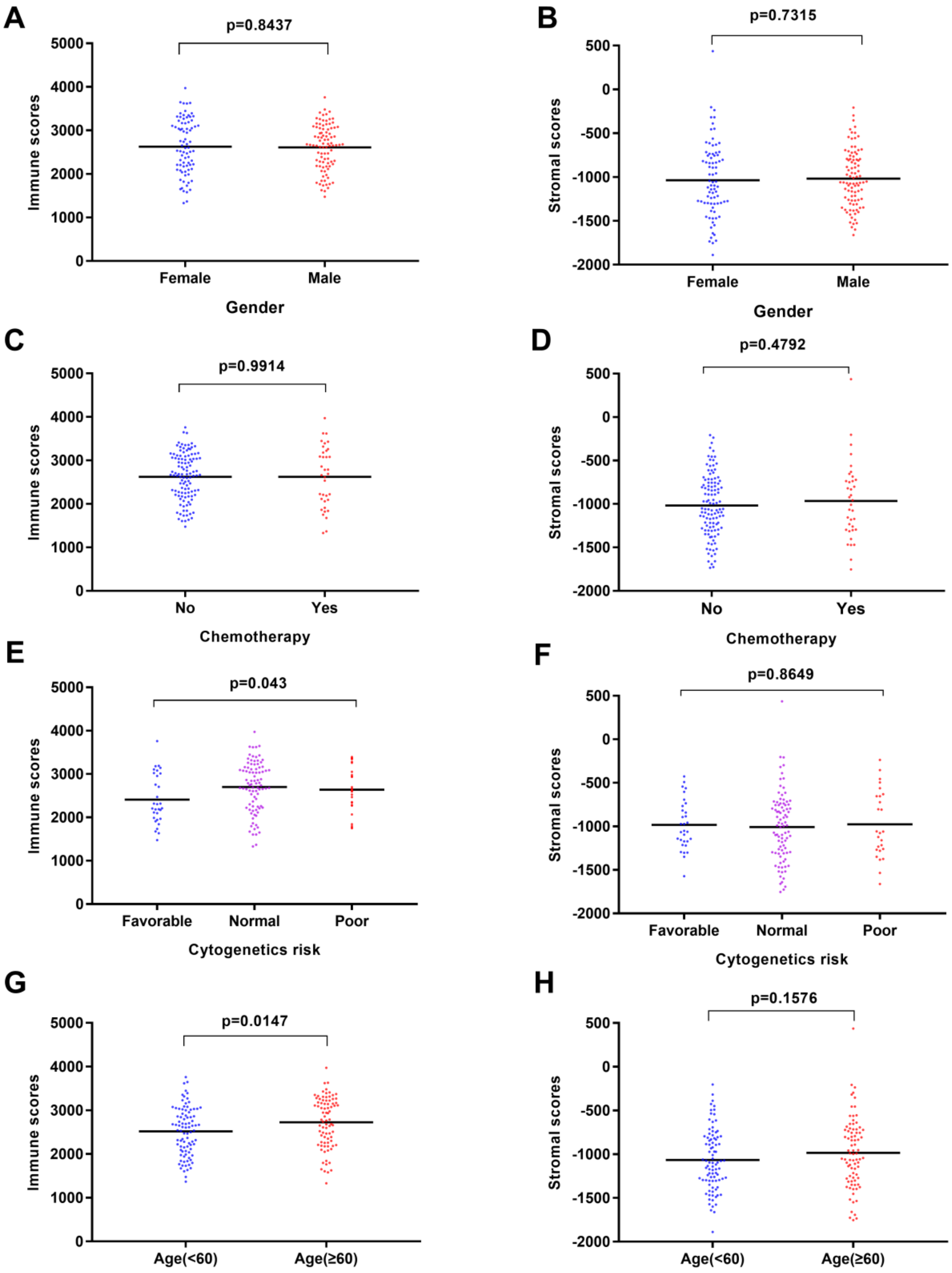

Supplementary Figure 1. Association of immune and stromal scores with clinical features of AML patients. (**A**) Distribution of immune scores based on gender (two-tailed t-test, p = 0.8437). (**B**) Distribution of stromal scores based on gender (two-tailed t-test, p = 0.7315). (**C**) Distribution of immune scores based on history of chemotherapy (two-tailed t-test, p = 0.9914). (**D**) Distribution of stromal scores based on history of chemotherapy (two-tailed t-test, p = 0.4792). (**E**) Distribution of immune scores based on cytogenetic risk categories (two-tailed t-test, p = 0.043). (**F**) Distribution of stromal scores based on cytogenetic risk categories (two-tailed t-test, p = 0.8649). (**G**) Distribution of immune scores based on patient's age (two-tailed t-test, p = 0.0147). (H) Distribution of stromal scores based on patient's age (two-tailed t-test, p = 0.1576).

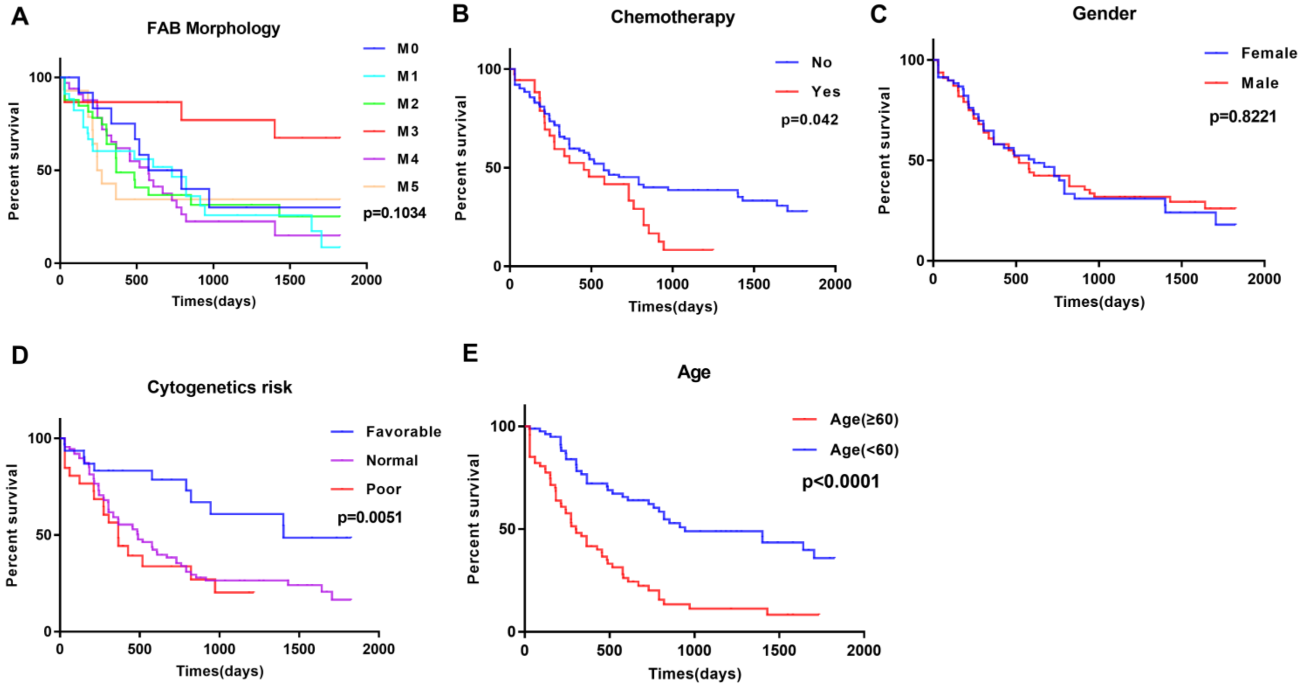

Supplementary Figure 2. Overall survival of AML patients according to clinical features. (**A**) FAB morphological subtypes (log rank test, $p = 0.1034$). (**B**) History of chemotherapy (log rank test, $p = 0.042$). (**C**) Patient's gender (log rank test, $p = 0.8221$). (**D**) Cytogenetic risk categories (log rank test, $p = 0.0051$). (E) Patient's age (log rank test, $p < 0.0001$).

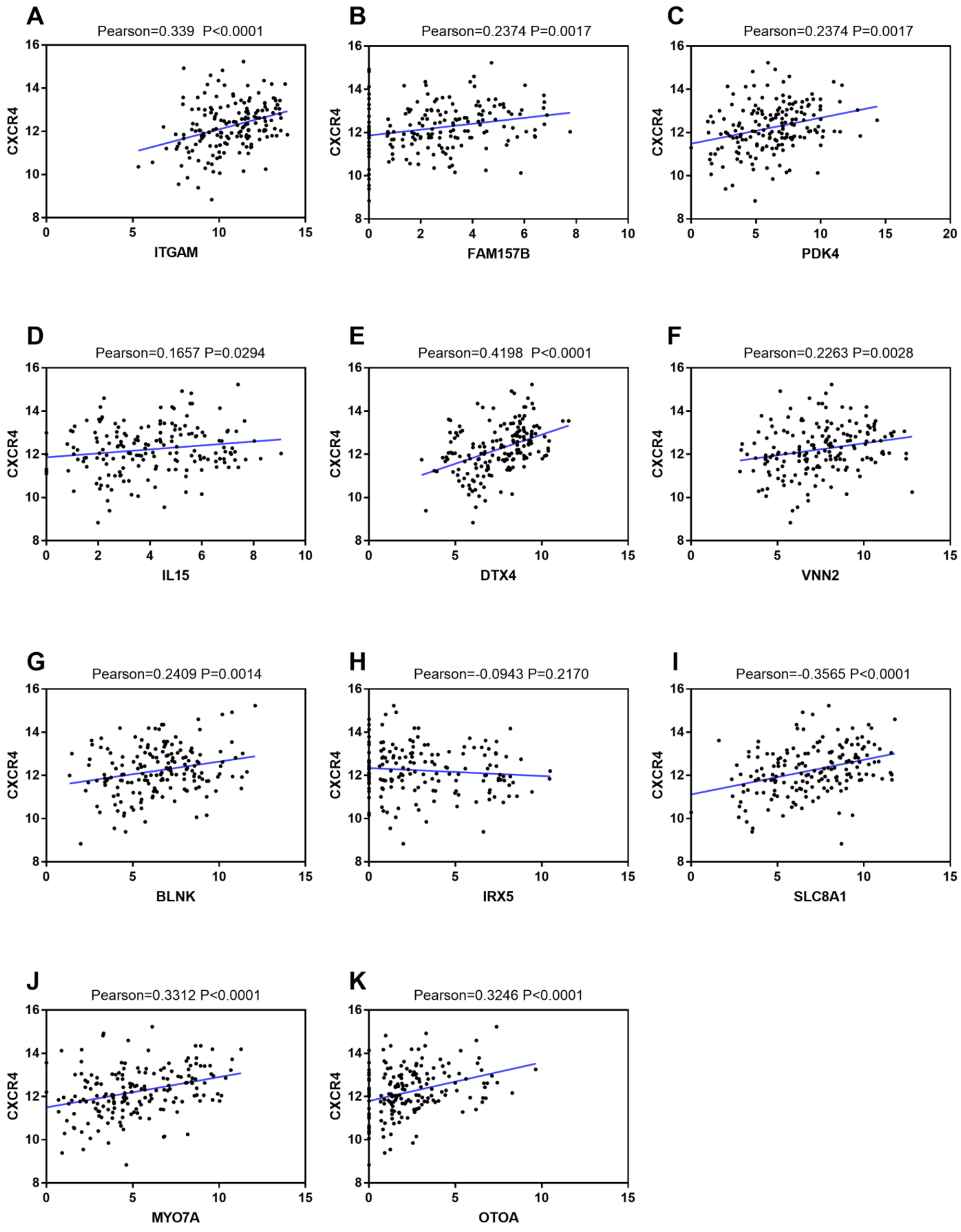

Supplementary Figure 3. Pearson's correlation between the 11 validated genes and CXCR4 expression ($p < 0.05$).

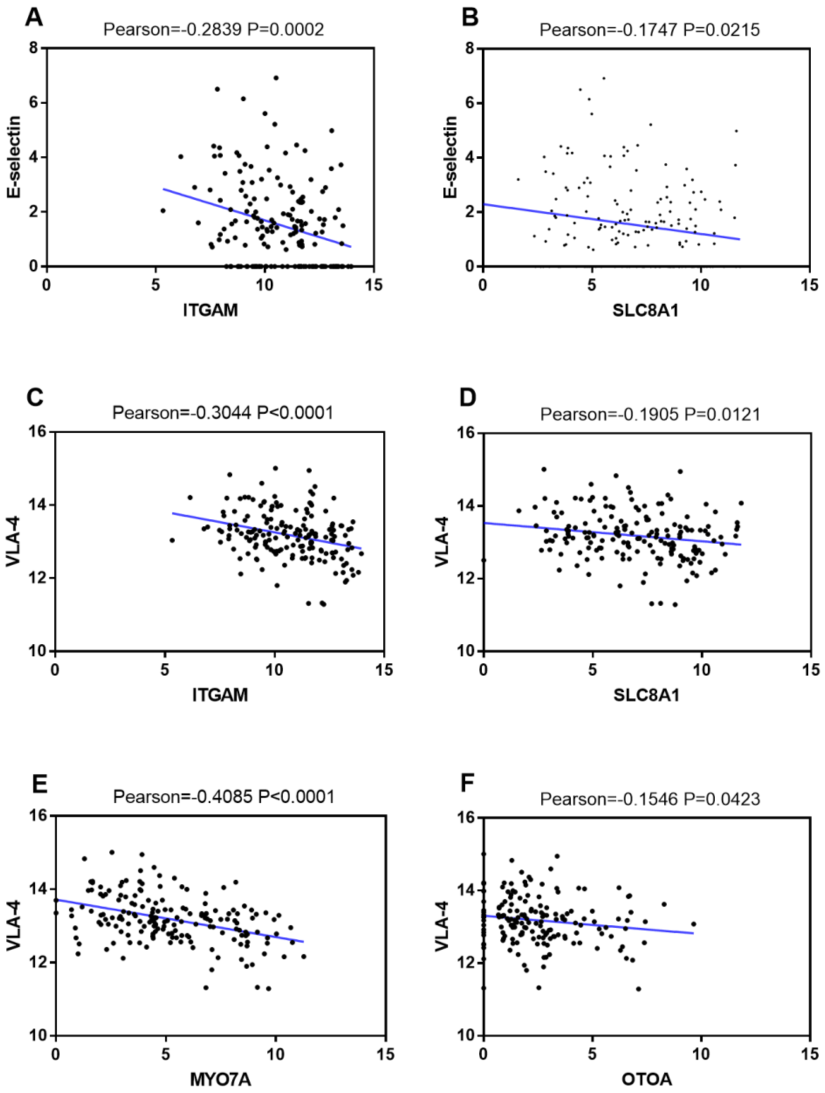

Supplementary Figure 4. Pearson's correlation between the 11 validated genes and E-selectin and VLA-4 expression ($p < 0.05$).
