# Supplementary material for: Identification of prognostic genes in the acute myeloid leukemia microenvironment
Source: Aging (Albany NY). 2019 Nov 18;11(22):10557–80. doi: 10.18632/aging.102477 (PMC6914404; doi:10.18632/aging.102477)
Supplement: Supplementary Tables [file aging-11-102477-s002..pdf]

SUPPLEMENTARY TABLES

Supplementary Table 1. Top 20 hub genes analyzed from the protein-protein interaction network of the DEGs (ranked by the degree score of each node).

Gene	Degree	MCODE_Cluster	MCODE_Score	Betweenness	Closeness
C1QB	16	Cluster 1	13.18965517	11.16473526	0.666666667
CCR1	26	Cluster 1	15.26515152	51.87278832	0.842105263
CCR5	27	Cluster 1	13.57957958	56.81499611	0.864864865
CD14	19	Cluster 2	10.96969697	41.98415551	0.650793651
CD163	23	Cluster 1	13.39772727	22.6970807	0.780487805
CD68	24	Cluster 1	15.96969697	23.39919525	0.8
CD86	23	Cluster 2	11.93236715	56.52134607	0.672131148
CYBB	21	Cluster 1	13.39772727	38.88020313	0.744186047
FCER1G	17	Cluster 1	14.98418972	30.85424298	0.680851064
FCGR2A	19	Cluster 2	11.3820598	34.36167962	0.650793651
FCGR2B	26	Cluster 1	13.82051282	46.86588412	0.842105263
FCGR3A	23	Cluster 2	11.83170732	26.6072905	0.694915254
HCK	25	Cluster 2	12.52462121	44.00774923	0.719298246
IL10	23	Cluster 1	13.47593583	27.72947608	0.780487805
IRF8	15	Cluster 2	11.58064516	8.442863115	0.602941176
ITGAM	35	Cluster 2	11.33526851	328.0639929	0.872340426
LILRB2	29	Cluster 2	12.65714286	68.60339587	0.773584906
MNDA	27	Cluster 2	12.75913978	55.64109135	0.745454545
TLR7	15	Cluster 2	11.3562753	27.78341616	0.611940299
TLR8	32	Cluster 2	10.23796933	119.978408	0.82

Note: Genes that marked yellow predicted poor overall survival in acute myeloid leukemia patients.

Supplementary Table 2. DEGs whose expression predicted poor overall survival in AML patients (log rank test, $p < 0.05$).
