# Supplementary material for: Identification of prognostic genes in the acute myeloid leukemia microenvironment
Source: Aging (Albany NY). 2019 Nov 18;11(22):10557–80. doi: 10.18632/aging.102477 (PMC6914404; doi:10.18632/aging.102477)
Supplement: Supplementary Table 2 [file aging-11-102477-s001..doc]

**Supplementary Table 2. The DEGs whose expression predicted significant poor overall survival in AML patients. (Log rank test, p < 0.05)**

| **No.** | **Gene** | **Log rank test p value** |
| --- | --- | --- |
| 1 | ABI3 | 0.0014 |
| 2 | ADAMDEC1 | 0.0071 |
| 3 | ADAP2 | 0.0283 |
| 4 | ADM | 0.0134 |
| 5 | ALOX5AP | 0.0090 |
| 6 | AMICA1 | 0.0187 |
| 7 | BCL2A1 | 0.0001 |
| 8 | BLNK | 0.0178 |
| 9 | C17orf87 | 0.0418 |
| 10 | C20orf118 | 0.0132 |
| 11 | C7orf58 | 0.0017 |
| 12 | CASP5 | 0.0341 |
| 13 | CCR1 | 0.0084 |
| 14 | CCR5 | 0.0271 |
| 15 | CD163 | 0.0046 |
| 16 | CD1D | 0.0285 |
| 17 | CD1E | 0.0183 |
| 18 | CD300C | 0.0104 |
| 19 | CD300LB | 0.0387 |
| 20 | CDC42EP1 | 0.0438 |
| 21 | CLEC10A | 0.0306 |
| 22 | CLEC4A | 0.0224 |
| 23 | CLEC7A | 0.0435 |
| 24 | CMKLR1 | 0.0098 |
| 25 | CRTAM | 0.0131 |
| 26 | CTSS | 0.0485 |
| 27 | CUEDC1 | 0.0032 |
| 28 | CX3CR1 | 0.0242 |
| 29 | CXCL10 | 0.0180 |
| 30 | CXCL16 | 0.0290 |
| 31 | CYP27A1 | 0.0099 |
| 32 | DTX4 | 0.0172 |
| 33 | EPB41L3 | 0.0075 |
| 34 | EPS8 | 0.0267 |
| 35 | FAM129B | 0.0118 |
| 36 | FAM157B | 0.0335 |
| 37 | FCGR2B | 0.0076 |
| 38 | FCGR2C | 0.0227 |
| 39 | FGD2 | 0.0030 |
| 40 | FGR | 0.0366 |
| 41 | FXYD6 | 0.0092 |
| 42 | GIMAP8 | 0.0017 |
| 43 | GNGT2 | 0.0006 |
| 44 | GPBAR1 | 0.0059 |
| 45 | HFE | 0.0290 |
| 46 | HK3 | 0.0273 |
| 47 | HLA.DQB1 | 0.0173 |
| 48 | HLA.DRB6 | 0.0414 |
| 49 | HSPA6 | 0.0241 |
| 50 | HSPA7 | 0.0075 |
| 51 | HTR7 | 0.0003 |
| 52 | IFI30 | 0.0055 |
| 53 | IL10 | 0.0315 |
| 54 | IL10RA | 0.0049 |
| 55 | IL15 | 0.0035 |
| 56 | IL1R2 | 0.0010 |
| 57 | IRX5 | 0.0148 |
| 58 | ITGAM | 0.0191 |
| 59 | KCNE1 | 0.0271 |
| 60 | KCNMB1 | 0.0003 |
| 61 | KCNQ1 | 0.0023 |
| 62 | KIAA0513 | 0.0086 |
| 63 | LGALS2 | 0.0212 |
| 64 | LILRA1 | 0.0053 |
| 65 | LILRA3 | 0.0227 |
| 66 | LILRA4 | 0.0354 |
| 67 | LILRA5 | 0.0063 |
| 68 | LILRA6 | 0.0473 |
| 69 | LILRB1 | 0.0007 |
| 70 | LILRB2 | 0.0103 |
| 71 | LY86 | 0.0252 |
| 72 | LY96 | 0.0006 |
| 73 | MRC1 | 0.0012 |
| 74 | MS4A4A | 0.0095 |
| 75 | MSR1 | 0.0214 |
| 76 | MYBPH | 0.0280 |
| 77 | MYO7A | 0.0027 |
| 78 | MYOF | 0.0406 |
| 79 | NAPSB | 0.0162 |
| 80 | NCF1 | 0.0376 |
| 81 | NCF1B | 0.0199 |
| 82 | NCF1C | 0.0258 |
| 83 | NRGN | 0.0023 |
| 84 | OAS1 | 0.0054 |
| 85 | OTOA | 0.0451 |
| 86 | PDK4 | 0.0348 |
| 87 | P2RY13 | 0.0499 |
| 88 | POU2F2 | 0.0133 |
| 89 | PTAFR | 0.0452 |
| 90 | RPGRIP1 | 0.0444 |
| 91 | S100A9 | 0.0306 |
| 92 | SECTM1 | 0.0351 |
| 93 | SERPINB2 | 0.0326 |
| 94 | SIGLEC7 | 0.0115 |
| 95 | SIGLEC9 | 0.0439 |
| 96 | SLC15A3 | 0.0009 |
| 97 | SLC26A11 | 0.0391 |
| 98 | SLC8A1 | 0.0009 |
| 99 | SLC9A9 | 0.0146 |
| 100 | SULF2 | 0.0343 |
| 101 | THBS1 | 0.0144 |
| 102 | TLR7 | 0.0275 |
| 103 | TLR8 | 0.0156 |
| 104 | TMEM132A | 0.0040 |
| 105 | TNFRSF11A | 0.0425 |
| 106 | TNFRSF1B | 0.0027 |
| 107 | TNNI2 | 0.0071 |
| 108 | VCAN | 0.0258 |
| 109 | VENTX | 0.0299 |
| 110 | VNN1 | 0.0113 |
| 111 | VNN2 | 0.0067 |
| 112 | VNN3 | 0.0069 |
